# Identification of viral protease-dependent cleavage sites within the human astrovirus polyprotein

**DOI:** 10.1128/jvi.01321-24

**Published:** 2024-12-04

**Authors:** Samaneh Mehri, Brooke Bengert, Madeline Holliday, Lochlain Corliss, Peter E. Prevelige, Nicholas J. Lennemann

**Affiliations:** 1 Department of Microbiology, University of Alabama at Birmingham 155591 , Birmingham, Alabama, USA; University of Michigan Medical School, Ann Arbor, Michigan, USA

**Keywords:** astrovirus, positive strand RNA virus, 3C-like protease, serine protease, polyprotein, protease

## Abstract

Infection by human astrovirus (HAstV), a small, positive-strand RNA virus, is a major cause of gastroenteritis and has been implicated in an increasing number of severe, sometimes fatal, neurological diseases since 2008. Currently, there are no vaccines or antiviral treatments available to treat HAstV infection. An attractive target for antiviral therapeutics is the viral protease due to its essential functions throughout infection. However, the molecular mechanisms of the HAstV protease, nonstructural protein 1a/3 (nsp1a/3), are poorly understood. In fact, the specific residues within the cleavage junctions that are targeted by nsp1a/3 during polyprotein processing have yet to be experimentally identified. To identify the junctions between viral proteins, we performed mass spectrometry and site-directed mutagenesis using epitope-tagged viral polyprotein expression plasmids. Using these strategies, we identified a consensus motif that is found throughout the polyprotein near previously proposed junctions. We found that cleavage occurs after a hydrophobic residue – X – Gln motif. Further mutagenesis of surrounding sequence identified the importance of basic residues following the motif for efficient processing. Cleavage at each junction was determined to be essential for the production of progeny virions. However, abolishing nsp1a/4-VPg cleavage allowed efficient replication, suggesting that VPg can function in an intermediate form. Overall, our results identify a conserved cleavage motif that is recognized by the nsp1a/3 protease within the viral polyprotein, and cleavage at this motif was found to be essential for the recovery of progeny virions. These findings will be instrumental in further understanding the basic functions of HAstV polyprotein processing during infection.

**IMPORTANCE:**

Human astroviruses (HAstVs) are a leading cause of non-bacterial gastroenteritis in children, elderly individuals, and immunocompromised patients. However, infection by divergent strains of HAstV is now recognized as a causative agent of severe neurological diseases, which can have fatal outcomes. Despite the global prevalence of HAstV, we currently have a limited understanding of the biology of these viruses. Translation of the viral genome leads to the production of polyproteins that are processed by viral and host proteases into functional proteins. In this study, we identified a conserved recognition sequence targeted by the viral protease for cleavage. Importantly, these findings elucidate the N- and C-termini of the nonstructural proteins within the HAstV polyprotein, offering valuable information for future studies on the function of individual viral proteins. Similar to other positive-sense RNA viruses, the necessity of proteolytic processing for the HAstV polyprotein highlights the viral protease as a promising target for antiviral development.

## INTRODUCTION

Astroviruses are small, non-enveloped, positive-sense single-stranded RNA (+ssRNA) viruses in the Astroviridae family within the *Mamastrovirus* (MAstV) genus (1–3). Human astroviruses (HAstVs) are classified into three divergent groups, the classical genotype and two noncanonical genotypes, MLB and VA1 (4–7). Classical strains are now recognized as a leading cause of nonbacterial gastroenteritis in children, the elderly, and immunocompromised individuals (5, 8, 9). In 2008 and 2009, two novel strains were discovered that have been associated with neurological diseases, which can be fatal (6, 7, 10–14). These strains are more closely related to non-human astroviruses that cause severe disease in other mammals, including ovine and mink strains, which suggests there may be recombination events between strains (9, 10, 15–17). There are currently no antiviral treatments for HAstVs, partially due to a poor understanding of the molecular virology and cellular biology of infection. The prevalence and continuous emergence of new astroviruses with the potential for zoonotic transmission necessitate rigorous study of these pathogens (2, 3, 6, 14, 18, 19).

**Fig 1:**
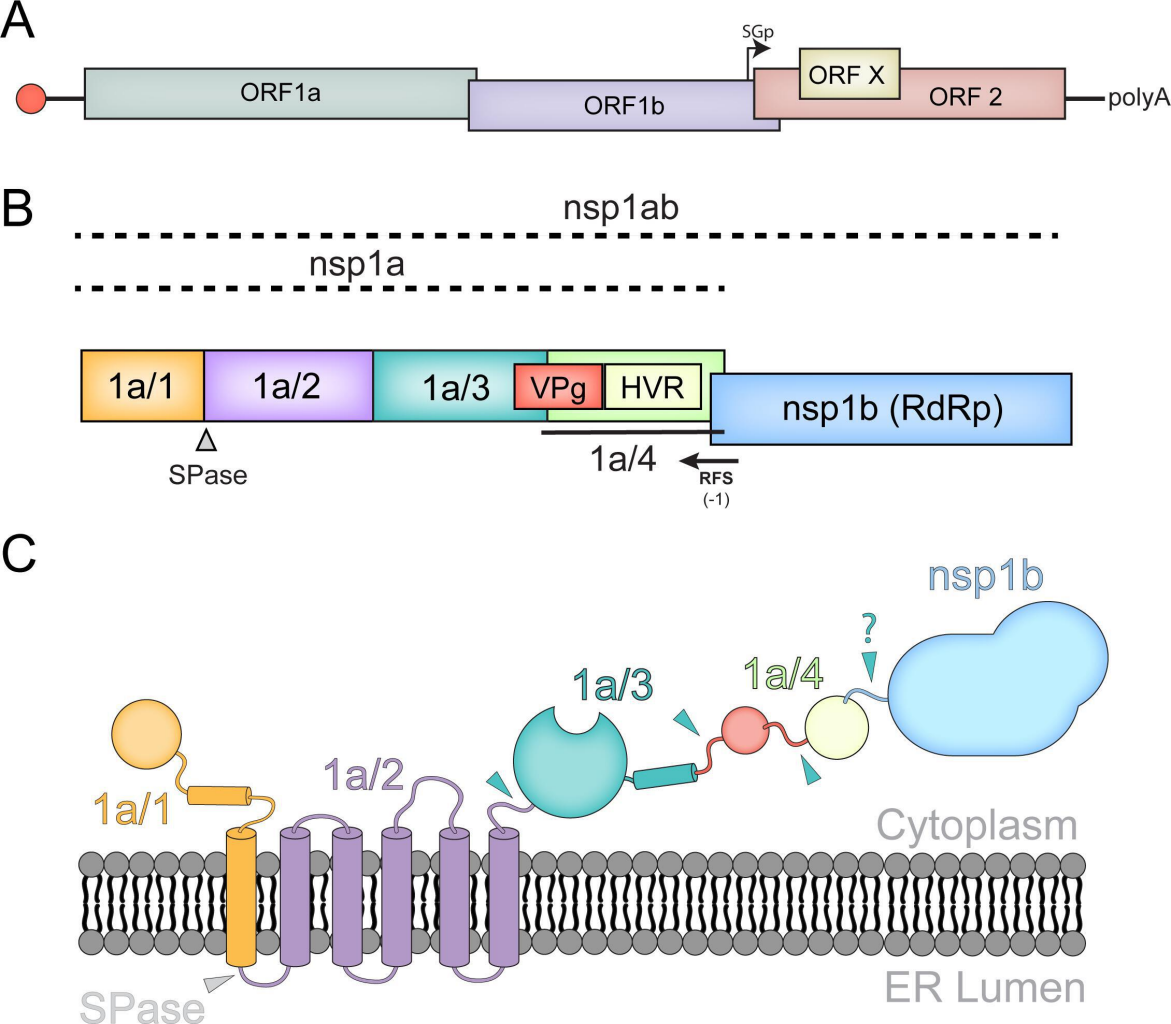
HAstV genome and polyprotein organization. (**A**) HAstV genome organization, VPg, red circle; SGp, subgenomic promoter. VPg-linked genomic RNA includes four ORFs (ORF1a, ORF1b, ORFX, and ORF2). (**B**) Linear model for nsp1a and nsp1ab polyproteins showing previously predicted protein products. (**C**) Predicted model of nsp1ab polyprotein topology showing host protease cleavage site (gray) and predicted nsp1a/3-dependent cleavage sites (cyan).

The ~6.8 Kb genome contains three open reading frames (ORF1a, ORF1b, and ORF2) encoding both nonstructural (nsp1a and nsp1ab) and structural polyproteins (1). Additionally, classical HAstVs and MLBs contain an alternative start codon within ORF2 that leads to production of ORFX, which encodes a viroporin (Fig. 1A and B) (20). The nsp1a polyprotein contains an N-terminal protein (nsp1a/1), a highly hydrophobic protein (nsp1a/2), a 3C-like serine protease (nsp1a/3), and nsp1a/4 that is proposed to be further processed into a viral protein linked to the genome (VPg) and hypervariable region (HVR; Fig. 1B) (21). A (−1) ribosomal frameshift (RFS) during the translation of the C-terminus of nsp1a/4 results in the co-translation of ORF1b, which contains the viral RNA-dependent RNA polymerase (RdRp, nsp1b) (22–24). The functions of many of these proteins and the presence of functional intermediates remain unknown due to the lack of information on the mechanisms of polyprotein processing.

HAstV polyprotein processing is mediated by both viral and host proteases. The cleavage between nsp1a/1 and nsp1a/2 occurs independently of the viral protease and is predicted to be performed by a host signal peptidase (25). The remaining cleavage events within nsp1a are mediated by the viral protease, nsp1a/3 (25). However, it is unknown if nsp1a/3 activity is required for the cleavage between nsp1a and nsp1b (Fig. 1C). Previous studies predicted that nsp1a cleavage events occur between Val 409/Ala 410, Gln 567/Thr 568, and Glu 654/Ile 655; however, these predictions are solely based on the size of protein bands visible on immunoblots and have not been experimentally validated (21, 25–27). Thus, further investigation is needed to identify nsp1a/3-dependent cleavage sites within the polyprotein.

Overall, these studies highlight the lack of information regarding HAstV protease activity and polyprotein processing, for which thorough investigation has been limited due to the absence of tractable molecular tools. Thus, we sought to develop genetic tools to advance our understanding of nsp1a/3-mediated HAstV1 polyprotein processing. Moreover, identification of a cleavage recognition motif by nsp1a/3 will define the N- and C-termini of each nonstructural protein within the HAstV polyprotein, which will allow for better determination of the functions for individual viral proteins. Given the essential roles of viral proteases during +ssRNA virus infection, HAstV nsp1a/3 is an attractive target for antiviral development. However, the lack of mechanistic knowledge related to this protein complicates attempts to design targeted therapeutics. Here, we identify and confirm nsp1a/3-dependent cleavage sites at a conserved target sequence found at nonstructural protein junctions within the viral polyprotein.

## MATERIALS AND METHODS

### Cell culture

Cells were cultured at 37°C and 5% CO_2_. HEK-293T cells (ATCC, CRL-11268) were maintained in Dulbecco’s modified Eagle’s medium supplemented with 10% fetal bovine serum (FBS) and 100 IU penicillin/100 µg/mL streptomycin. Human colorectal adenocarcinoma cells (Caco2, ATCC HTB-37) and baby hamster kidney cells (BHK-21 clone 15; a generous gift from Dr. Douglas Brackney, Connecticut Agricultural Experiment Center) were maintained in modified Eagle’s medium supplemented with 10% FBS, 10% Sodium Pyruvate, 10% non-essential amino acids, and 100 IU penicillin/100 µg/mL streptomycin. Human hepatoma cells (Huh7) were maintained in Dulbecco’s modified Eagle’s medium supplemented with 10% FBS and 100 IU penicillin/100 µg/mL streptomycin and 9 g/L glucose. Hybridoma cells (ATCC, CRL-8795), secreting monoclonal antibody 8E7 (mouse anti-HAstV1 capsid protein), were grown in Iscove’s modified Dulbecco’s medium with 2 mM L-glutamine and 15% FBS.

### Plasmids

Vectors used for fusion/epitope tagging were generated in pcDNA3.1 by insertion of green fluorescent protein (GFP) between HindIII and BamHI restriction sites and V5- or HA-epitope tags between EcoRI and XhoI restriction sites. These vectors were linearized with BamHI and used in HiFi Assembly (NEB) with DNA encoding viral proteins. The nsp1a∆655 sequence was synthesized as a gBlock (Integrated DNA Technologies). Cloning of pcDNA3.1-GFP-nsp1a-V5 and pcDNA3.1-GFP-nsp1ab-V5 was accomplished via PCR amplification using cDNA generated from HAstV1-infected Caco2 cells with the following primers: nsp1a_F (5′-GCTTGGTACCGAGCTCGGATCCATGGCACACGGTGAGCC-3′) with nsp1a_R (5′-GAATTCCACCACACTGGAATGAGTGGTAATTTTGGG-3′) or nsp1b_R (5′GAATTCCACCACACTGGAGCCATCACACTTCTTTGG-3′).

### Site-directed mutagenesis

Alanine substitutions in plasmids encoding viral nonstructural proteins were generated using a modified site-directed mutagenesis protocol. Site-specific forward and reverse primers were used with nsp1a_F, nsp1a∆655_R, nsp1a_R, or nsp1b_R primers to generate overlapping PCR fragments. These fragments were assembled into pcDNA3.1-GFP-V5 linearized with BamHI using HiFi Assembly (NEB). Site-specific primer pair sequences used were nsp1a∆655_R (5′- GAATTCCACCACACTGGAGATTTCATCACGCAGCAC-3′), S551A (5′-CAGGACGGGATGGCGGGTGCACCAGTTTG-3′ and 5′-CAAACTGGTGCACCCGCCATCCCGTCCTG-3′), V409A (5′-ACTAGGATCAAAAATGCTGCATTTGACTTCTTC-3′ and 5′-GAAGAAGTCAAATGCAGCATTTTTGATCCTAGT-3′), Q567A (5′-AGTCCATGCTGCTAACACTGGGTATACTGGAGG-3′ and 5′-AGTGTTAGCAGCATGGACTGCTAACACCCGAC-3′), E654A (5′-AAGGTGCTGCGTGATGCCATCAATGGAATACTT-3′ and 5’- AAGTATTCCATTGATGGCATCACGCAGCACCTT-3′), QK415A (5′-ACTTCTTCGCAGCACTGAAGCAGTCAGGGGTGCG-3′ and 5′-CTTCAGTGCTGCGAAGAAGTCAAATGCAAC-3′), Q415A (5′-TTCTTCGCAAAGCTGAAGCAGTCAGGGG-3′ and 5′-CAGCTTTGCGAAGAAGTCAAATGCAAC-3′), K416A (5′-TTCCAGGCACTGAAGCAGTCAGGGGTGCG-3′ and 5’- GCTTCAGTGCCTGGAAGAAGTCAAATGCA-3′), Q628A (5′-AGTGTTGCACTAGAAGCGAAAAGTGTCAGCGA-3′ and 5’- TCGCTGACACTTTTCGCTTCTAGTGCAACACT-3′), Q664A (5′-CCATTCCTAGCAAAAAAGAAAGGCAAGACCAAG-3′ and 5’- TTTCTTTTTTGCTAGGAATGGTGCAAGTATTC-3′), Q755A (5′-AACTTTGACGCAgGAAAACCAATTCCTGCCC-3′ and 5’- TGGTTTTGCTGCGTCAAAGTTAATTACTTC-3′), Q894A (5′-GGTTTCCTCGCAAGGTTAAATCAAAAAAC-3′ and 5′-GATTTAACCCTTGCGAGGAAACCTTCCAAACC-3′), K665-667A (5′-CCATTCCTACAAGCAGCAGCAGGCAAGACCAAGCAT-3′ and 5′-GGTCTTGCCTGCTGCTGCTTGTAGGAATGGTGCAAG-3′), K665A (5’- CATTCCTACAAGCAAAGAAAGGCAAGACCAAG-3′ and 5’- GCCTTTCTTTGCTTGTAGGAATGGTGCAAGTATTC-3′), K666A (5′-CCATTCCTACAAAAAGCAAAAGGCAAGACCAAGCAT-3′ and 5′-GGTCTTGCCTTTTGCTTTTTGTAGGAATGGTGCAAG-3′), K667A (5′-CCATTCCTACAAAAAAAGGCAGGCAAGACCAAGCAT-3′ and 5’- CTTGGTCTTGCCTGCCTTTTTTTGTAGGAATGGTGCAAG-3′), F662A (5′-CTTGCACCAGCACTACAAAAAAAGAAAGGC-3′ and 5′-TTTTTGTAGTGCTGGTGCAAGTATTCCATTG-3′), and L663A (5′-GCACCATTCGCACAAAAAAAGAAAGGCAAG-3′ and 5’- CTTTTTTTGTGCGAATGGTGCAAGTATTCC-3′).

### Immunoblots

HEK293T cells were transfected with polyethylenimine (PEI, 25 kDa) at a 1:1 ratio of DNA (μg) to 1 µL PEI (1 mg/mL stock). Protein from cell lysates was prepared using 1× radioimmunoprecipitation assay (RIPA) buffer + 0.1% SDS + protease inhibitor cocktail (Sigma, St. Louis, MO, USA). Lysates were clarified by centrifugation at 12,000 × *g* for 10 minutes prior to sample preparation for SDS-PAGE. Samples were prepared with 6× SDS loading buffer ± betamercaptoethanol (BME) ± boiling, as indicated, then separated on 4%–20% Tris-glycine polyacrylamide precast gels (BioRad). Protein was then transferred to nitrocellulose membranes followed by 30 minutes of blocking in 10% non-fat milk + phosphate buffered saline (PBS). Protein was detected using the following antibodies: mouse anti-V5 epitope tag (1:5,000, Invitrogen), rabbit anti-GFP (1:2,000, ProteinTech), rabbit anti-HA (1:2,000, Cell Signaling), rabbit anti-glyceraldehyde-3-phosphate dehydrogenase (GAPDH; 1:2,000, ProteinTech), and corresponding near-infrared dye-conjugated secondary antibodies (LiCor) diluted in PBS + 0.01% Tween-20 + 5% non-fat milk. Immunoblots were imaged using an Odyssey CLx imaging system and ImageStudio software (LiCor). Immunoblots throughout the manuscript are representative of three individual experiments.

### Immunoprecipitation

HEK293T cells were transfected as previously described with GFP-nsp1a∆655-V5 expression plasmid. At 48 hours post transfection, cells were harvested in Hanks’ balanced salt solution (HBSS) complemented with protease inhibitor cocktail (Sigma, St. Louis, MO, USA) and 1% glucose (HBSS++). Cells were lysed by freezing in liquid nitrogen, thawing on ice, and sonicating on ice. Debris was removed from lysates by centrifugation at 4°C and 12,000 × *g* for 30 minutes. Clarified lysates were incubated with magnetic V5 trap-beads (Chromo Tek) for 3 hours at 4°C followed by washing 8× with HBSS++. After the last wash, protein on beads was eluted with 0.1% formic acid followed by incubation at 100°C for 10 minutes and stored at −20°C.

### Mass spectrometry analysis

Masses of purified protein on precipitated V5-trap beads were determined using a Waters Synapt G2-S(i) electrospray ionization time-of-flight (ESI-ToF) mass spectrometer equipped with a Waters Acquity liquid chromatography system. Five microliters each of either mock or transfected sample in 0.1% formic acid was loaded onto a Waters C18 reverse phase column (130 Å, 1.7 µm, 2.1 × 50 mm). The column was developed at room temperature with a gradient of increasing acetonitrile. Data were collected in positive ion resolution mode to 3,000 m/z with Glu-Fib used as lockspray. The raw m/z data were transformed to mass data using the MaxEnt 1 algorithm in MassLynx 4.0. The observed unique mass was used with the MS-Nonspecific tool (UCSF, http://prospector2.ucsf.edu/) to identify peptide sequences derived from GFP-nsp1a∆655-V5 that contain the epitope tag at the end of the sequence.

### Multiple sequence alignment

Predicted cleavage sites within MAstV sequences were aligned to HAstV1 cleavage sites using ClustalW. The following sequences were used for analysis: MAstV1 (HAstV1 [NC_001943.1], HAstV2 [MK059950.1], HAstV3 [MG571777.1], HAstV4 [DQ070852.1], HAstV5 [MK059953.1], HAstV 6 [MK059954.1], HAstV7 [MK059955.1], and HAstV8 [MK059956.1]), MAstV2 (KF499111.1), MAstV3 (NC_025379.2), MAstV4 (MT451918.1), MAstV5 (KY765684.1), MAstV6 (JQ086552.1), MAstV8 (GQ415660.1), MAstV9 (NC_013060.1), MAstV10 (OL444939.1), MAstV13 (NC_002469.1), and MAstV18 (MW450837.1). A graphical representation of the consensus sequence based on the alignments was generated using WebLogo (28).

### Construction of DNA-launched infectious clone plasmid

The HAstV1 genome was isolated from stock virus, obtained through BEI Resources, NIAID, NIH: Human Astrovirus Type 1, Oxford, NR-51388, using PureLink Viral RNA/DNA Mini Kit (Invitrogen). Viral RNA was used to generate cDNA using SuperScript III Reverse Transcriptase and poly-dT primer (IDT), followed by PCR amplification using Q5 polymerase (NEB) and the following primers: pcAstV_F (5′-CGTTTAGTGAACCGGCCAAGAGGGGGGTGGTGATTG-3′) and pcAstV_R (5′-CCATGCCGGCCT_16_GCTTCTGATTAAATCAATTTTAAATGG-3′). The pcDNA6.2 vector was PCR amplified from pcDNA6.2-DENV 16681 (29), which contains a 5′ cytomegalovirus (CMV) promoter and 3′ hepatitis delta virus ribozyme (HDVr) sequence using the following primers: Vector_F (5′-GTTGAATCAACAGGTTCTGGCCGGCATGGTCCCAGCCTC-3′) and Vector_R (5′-CGTAGACTAACAACTCGGTTCACTAAACGAGCTCTGC-3′). The amplified DNA genome and vector were assembled using HiFi Assembly (NEB), followed by transformation into NEB Stable Competent *Escherichia coli* (NEB) at 30°C. The plasmid was purified by ZymoPURE Express Plasmid Midiprep Kit according to the manufacturer’s protocol, then sequenced using Plasmidsaurus.

Alanine substitutions were generated using a modified site-directed mutagenesis protocol. A 5′ PCR fragment was generated using 5′PstI_F (5′-GCGCAACGTTGTTGCCATTGCTGCA-3′) with a site-specific reverse primer (described above) and a 3′ fragment with at least 15 bp homology at the mutation site generated using a site-specific forward primer (described above) and 3′KpnI_R (5′-GATCTCTTTCTTAAGAAATAGG-3′). These fragments were assembled into pcDNA6.2-HAstV1 and digested with PstI (cut in the vector) and KpnI (cut in ORF1b) using HiFi Assembly (NEB). Plasmids with mutations were sequence verified using Plasmidsaurus.

### Recovery of virus from pcDNA-HAstV1

Virus was recovered by direct transfection of pcDNA-HAstV1 into Huh7 or Caco2 cells using PEI (25 kDa) at a 1:1 (vol/wt) ratio with plasmid. At 24 hours post transfection (hpt), cells were gently washed twice with DMEM-0 and replaced with serum-free Caco2 media containing 10 µg/mL porcine trypsin. At 72 hours post transfection, cells were subjected to three freeze/thaws in liquid nitrogen and 37°C water bath, respectively. Cell debris was removed by centrifugation at 5,000 × g for 5 minutes, and then, supernatants were collected for virus titration.

### Virus titration

Titrations were carried out as previously described (30). Caco2 cells were seeded in a 96-well plate with 2.5 × 10^4^ cells per well. The cells were grown for 3–4 days to allow for tight junction formation. Media was replaced with serum-free Caco2 media containing 0.3% bovine serum albumin (BSA) for an hour followed by infection with 10-fold dilutions of virus-containing supernatant. At 18 hours post infection, cells were fixed in PBS + 4% paraformaldehyde (PFA), permeabilized with PBS + 0.1% Triton X-100, washed with PBS, and incubated with primary mouse anti-HAstV1 capsid protein (8E7) 1:4 in PBS overnight at 4°C. Cells were then washed twice with PBS and incubated with 1:1,000 diluted goat anti-mouse Alexa Fluor 488, followed by two final PBS washes. Capsid positive foci were quantified using an Olympus IX83 inverted fluorescent microscope to calculate titer.

### Construction of pHAstV1rep-Duo reporter replicon

The subgenomic replicon was generated using HiFi Assembly (NEB) of five overlapping amplified fragments: pBR322 vector (Promega), T7 promoter followed by nucleotides 1–4,739 of HAstV1, a dual reporter protein (2A-NanoLuc-GFPzeo) in frame to the first 387 nucleotides of ORF2, and nucleotides 6,501–6,722 of HAstV1 with a polyA_16_. The vector was PCR amplified using Rep_vF (5′-GCTCGAGTCGAGCAATTCTTG-3′), which contains an XhoI site for plasmid linearization, and Rep_vR (5′-GTGAGTCGTATTAGCGGCCGCTCGACCGATGCCCTTGAGAGC-3′). The T7-1-4739nt fragment was amplified from pcDNA-HAstV1 using T7-HAstV_F (5′- GGGCATCGGTCGAGCGGCCGCTAATACGACTCACTATAGCCAAGAGGGGGGTGGTGATTG-3′) and 4739_R (5′-GGTAGCACCGCTGCCACCTAGCGCCTGCACAGGGCC-3′). The reporter insert was generated as two fragments, 2A-NanoLuc and GFPzeo, using PCR. The porcine teschovirus 2A skipping peptide was attached to the 5′ end of NanoLuciferase in two PCR reactions; the first reaction used 2A-NL_F1 (5′- AAAATCCCGGACCTGGATCCATGGTCTTCACACTCGAAGATTTC-3′) and NL_R (5′-CCCGCCGCTGCCACCACCTCCCGCCAGAATGCGTTCGCACAG-3′), which was then used in a second reaction to complete the 5′ 2A sequence using 2 A-NL_F2 (5′- GGCAGCGGTGCTACCAACTTCTCCCTGTTGAAGCAAGCTGGTGATGTAGAAGAAAATC-3′) and NL_R. The GFPzeo cassette was amplified from pDENVrep-GZ (31) (a generous gift from Dr. Carolyn Coyne, Duke University) using GFP_F (5′- GGTGGTGGCAGCGGCGGGATGGTGAGCAAGGGCGAGGAG-3′) and Zeo_R (5′- GCTATCCTTGTGGCACGCGTTTAGTCCTGCTCCTCGGCCAC-3′). The 6,501–6,722 + polyA_16_ was amplified from pcDNA-HAstV1 using 6501_F (5′-CGTAAATCAAGGAATGACAATG-3′) and 6722 pA_R (5′-CT_16_GCTTCTGATTAAATCAATTTTAAA-3′). After HiFi Assembly (NEB), the reaction was transformed into NEB Stable Competent *E. coli*. Plasmid sequence was verified using Plasmidsaurus.

Alanine substitutions were generated using a modified site-directed mutagenesis protocol, described above for pcDNA-HAstV1, but the forward primer used for generation of the 5′ fragments was T7-HAstV_F. Amplified fragments were assembled into the pHAstV1rep-Duo digested with NotI (cuts upstream of T7 promoter) and KpnI (cuts in ORF1b). Plasmids with mutations were sequence verified using Plasmidsaurus.

### *In vitro* transcription assay and viral RNA transfection

pHAstV1rep-Duo plasmids were linearized by XhoI enzyme. All RNAs were *in vitro* transcribed from the linearized plasmid using HiScribe T7 ARCA mRNA Kit (NEB) for 2 hours at 37°C, followed by DNase I digestion and LiCl precipitation.

### NanoLuciferase assay

BHK-21 cells were transfected with *in vitro* transcribed RNA of pHAstV1rep-Duo and mutants using TransIT-mRNA transfection kit (Mirus Bio) according to the manufacturer’s instructions. Cell lysates were prepared using 1× RIPA buffer + protease inhibitor cocktail (Sigma, St. Louis, MO, USA) at 0, 3, 6, 9, 12, and 24 hours post transfection. Cell debris was removed by centrifugation at 12,000 × *g* for 10 minutes. Luciferase activity was determined via Nano-Glo Luciferase Assay kit (Promega kit) according to the manufacturer’s instructions. Luminescence was measured using a BioTek Cytation 5 cell Imaging Multimode Reader.

### Image and data analysis

Figures and images were generated using Bio Render, WebLogo, Adobe Illustrator, Prism, and Adobe Photoshop. Predicted molecular weights were determined using Benchling.

## RESULTS

### Expression of tagged HAstV1 nonstructural polyproteins

**Fig 2:**
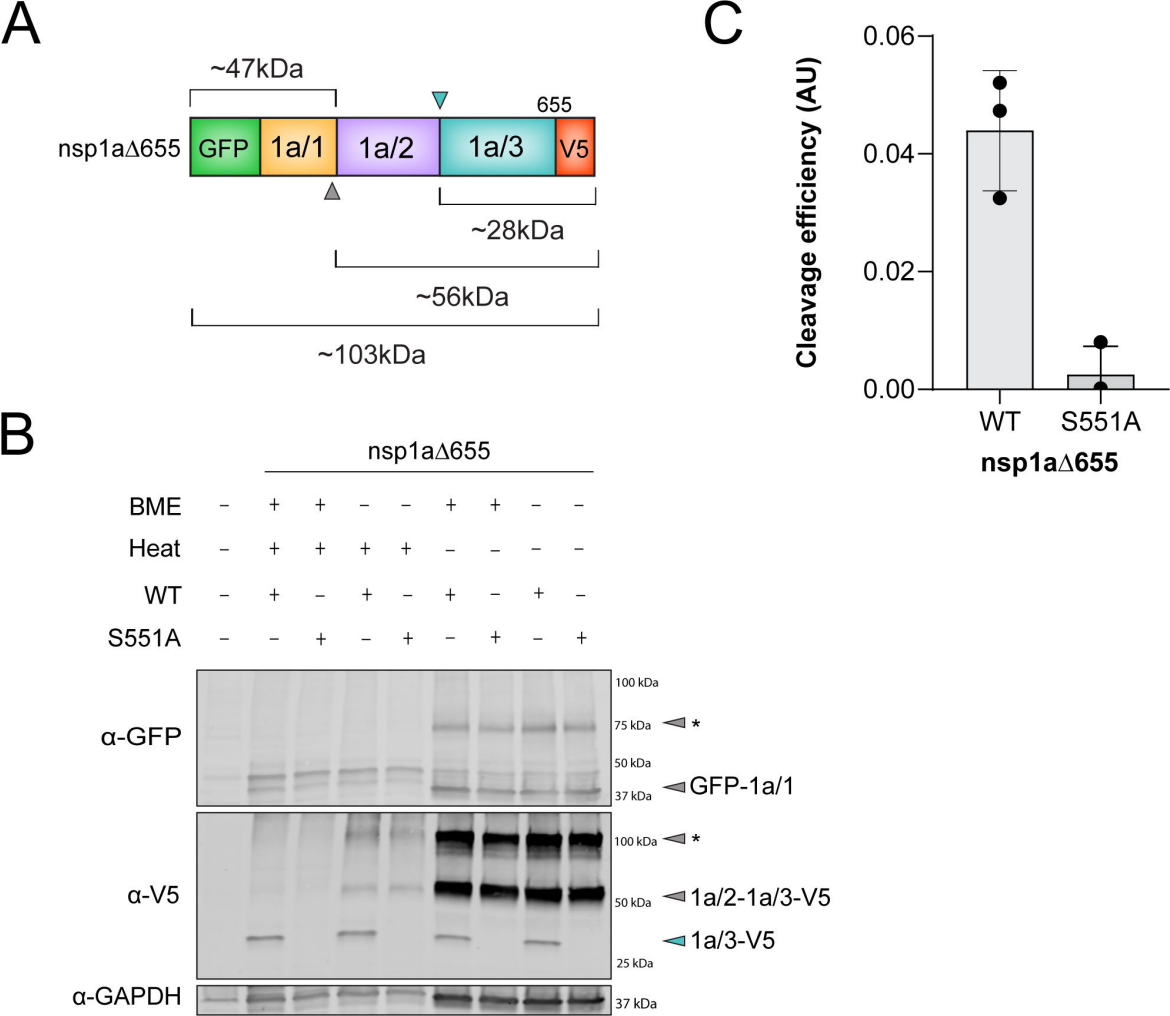
Expression of dual-tagged nsp1a∆655 nonstructural protein. (**A**) Linear model of dual-tagged nsp1a∆655 construct. Arrows indicate previously predicted cleavage sites targeted by host protease (gray) or nsp1a/3-dependent cleavage site (cyan), with corresponding sizes. (**B**) Representative immunoblot of lysates from HEK293T cells transfected with GFP-nsp1a∆655-V5 plasmid and the catalytically inactive protease (S551A) mutant plasmid ± BME ± heating conditions and probed for GFP, V5, and GAPDH. Annotated gray and cyan arrows represent predicted protein products based on molecular weight. * Gray arrows indicate potential aggregates or higher-order protein complexes due to sample preparation in the absence of heat. (**C**) Quantification of immunoblots represented in (**B**, lanes 6 and 7). Data represent the average ± SD intensity ratios in arbitrary units (AU) of the final cleavage product (cyan arrow) to full V5-signal within the lane, normalized to GAPDH intensity; individual data points of three independent experiments are shown in filled circles.

**Fig 3:**
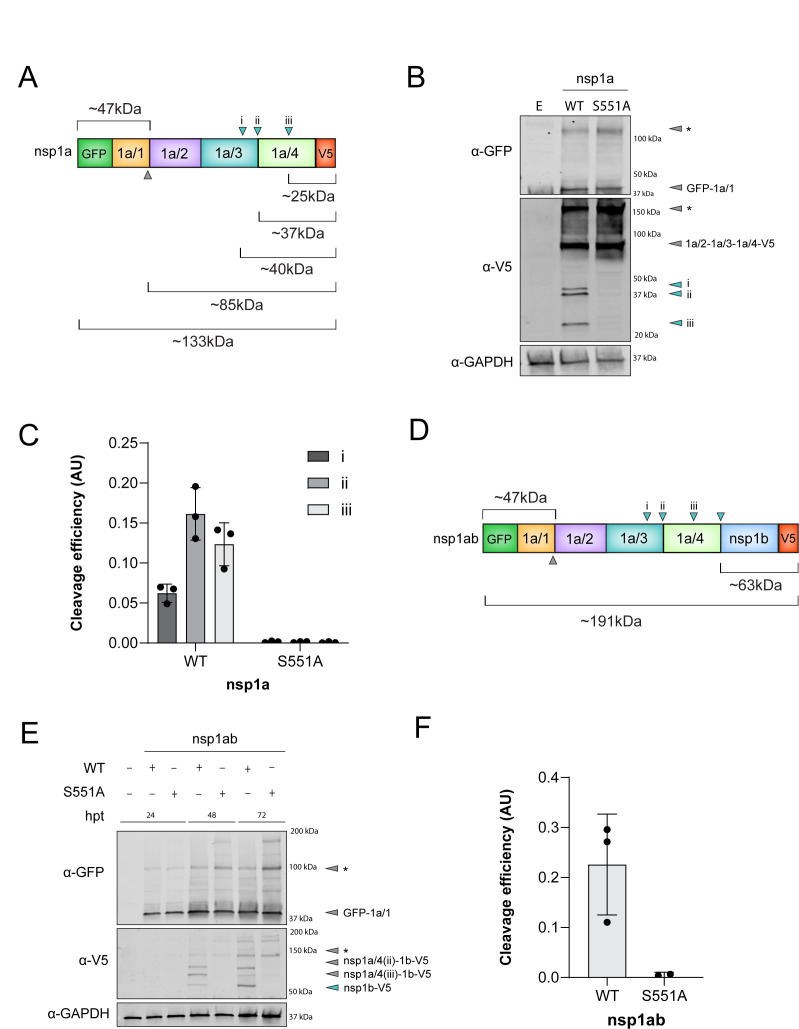
Expression of dual-tagged nsp1a and nsp1ab nonstructural proteins. (**A and D**) Linear models of dual-tagged polyprotein constructs. Arrows indicate previously predicted cleavage sites targeted by a host protease (gray) and nsp1a/3-dependent cleavage sites (cyan), with corresponding sizes. (**B and E**) Immunoblots of lysates from HEK293T cells transfected with either WT or S551A mutant (catalytically inactive) GFP-nsp1a-V5 expression plasmid at 24 hpt (**B**) or GFP-nsp1ab-V5 expression plasmid at the indicated time points post transfection (**E**) and probed for GFP, V5, and GAPDH. Gray and cyan annotated arrows represent predicted protein products based on molecular weight. * Gray arrows indicate potential aggregates or higher-order protein complexes due to sample preparation in the absence of heat. (**C and F**) Quantification of immunoblots represented in (**B**) and (E, 72 hpt). Data represent the average ± SD intensity ratios in arbitrary units (AU) of the final cleavage product (cyan arrow) to full V5-signal within the lane, normalized to GAPDH intensity; individual data points of three independent experiments are shown in filled circles.

Previous studies have identified viral proteins derived from HAstV polyproteins during infection or protein overexpression, which provided approximate molecular weights for each product (Table 1). These molecular weights have been used to predict polyprotein cleavage sites; however, these predictions have not been experimentally confirmed (21, 25, 27, 32–35). Thus, we sought to investigate the nsp1a/3-dependent HAstV1 nonstructural polyprotein cleavage events using a series of dual-tagged nonstructural polyprotein expression plasmids containing an N-terminal GFP and C-terminal V5-epitope tag (Fig. 2 and 3). To identify the first predicted nsp1a/3-dependent cleavage site between nsp1a/2 and nsp1a/3, we used a truncated nsp1a construct containing amino acids 1–655 (GFP-nsp1a∆655-V5), which was previously predicted to be the C-terminus of nsp1a/3 (21, 32) (Fig. 2A). Upon exogenous expression of GFP-nsp1a∆655-V5 in HEK 293T cells, we observed two major bands at ~47 and ~28 kDa via immunoblot of cell lysates, consistent with the predicted sizes for GFP-nsp1a/1 and nsp1a/3-V5, respectively (Fig. 2B) (26). Substitution of S551 for alanine in the nsp1a/3 catalytic triad resulted in the production of GFP-nsp1a/1, but not nsp1a/3-V5, indicating a lack of protease activity (Fig. 2B and C) (32). However, we were unable to efficiently observe the ~56 kDa (nsp1a/2–1a/3-V5) precursor that would be abundant upon loss of protease activity (Fig. 2B). Previous studies have shown that applying heat to membrane proteins can lead to aggregation and decrease detection via immunoblot (36–39). Thus, to optimize the detection of intermediate products, we altered the incubation conditions of lysates and found that boiling the lysates decreased the detection of non-cleaved intermediates, including nsp1a/2–1a/3-V5, which were observed in both wild-type (WT) and S551A non-heated lysates (Fig. 2B). Additionally, we observed a ~100 kDa band that was V5-positive and GFP-negative, which may represent a nsp1a/2–1a/3-V5 aggregate or a higher-order complex. Similarly, sample heating altered the migration of GFP-1a/1 and led to the appearance of a ~75 kDa band that may represent a higher-order complex. Given the importance of intermediate product detection, further cell lysate samples were treated with BME in the absence of boiling prior to separation via SDS-PAGE.

**TABLE 1:** Previously observed HAstV polyprotein products

Protein	MW (~kDa)	Reference(s)
nsp1a	101	25, 27
nsp1a/1	19	21, 25
nsp1a/2	27	27
nsp1a/3	28	27, 32
nsp1a/4	35	21
nsp1a/4-VPg	13–15	33 – 35
nsp1a/4-p20	20	21, 25, 27
nsp1ab	165	21, 25, 27
nsp1b	57	21

Next, we used a GFP-nsp1a-V5 expression plasmid to investigate cleavage between nsp1a/3 and nsp1a/4 (Fig. 3A). Again, we observed efficient production of GFP-nsp1a/1; however, we observed numerous nsp1a/4-V5-derived products, with prominent bands at full length (GFP-nsp1a-V5), ~85 kDa (nsp1a/2–4-V5), ~40 kDa (i), ~37 kDa (ii), and ~25 kDa (iii) (Fig. 3B). Mutagenesis of the catalytic serine in nsp1a/3 (S551A) blocked the detection of the major products (i), (ii), and (iii), suggesting that viral protease activity is required for their production. The ratio of cleaved products (i), (ii), and (iii) to total V5 signal in the lane indicates production of (i) is less efficient than production of (ii) and (iii) (Fig. 3C).

Lastly, we expressed GFP-nsp1ab-V5 to look at nsp1a/3-dependent production of nsp1b (Fig. 3D). Immunoblots of lysates from cells expressing this construct at 24 hpt resulted in minimal expression of the nsp1b product (Fig. 3E), likely due to the infrequency of the (−1) RFS required for translation of ORF1b (22, 24, 40). However, lysates from 48 and 72 hpt contain a prominent band at ~60 kDa, which was absent in the construct containing the S551A-inactivated protease, suggesting that the viral protease is responsible for the production of nsp1b (Fig. 3E and F). Together, these results confirm that nsp1a/3-dependent cleavage sites within nsp1a and nsp1ab can be investigated via overexpression of these polyprotein constructs.

### HAstV1 nsp1a/3 does not target Val 409, Gln 567, and Glu 654 for cleavage

**Fig 4:**
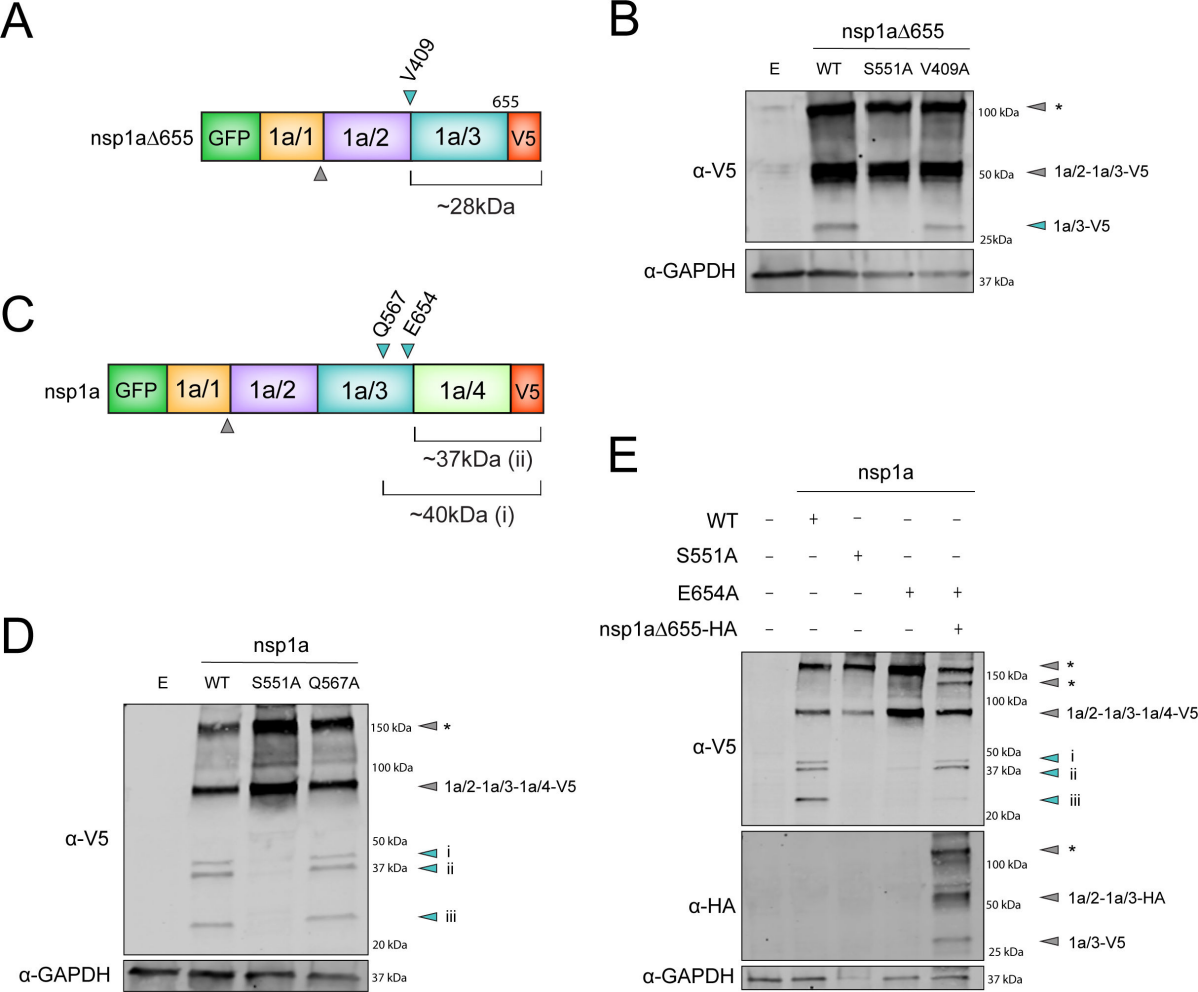
Analysis of previously predicted cleavage sites Val 409, Gln 567, and Glu 654. (**A and C**) Linear models of dual-tagged polyprotein constructs with indication of the previously predicted host protease cleavage site (gray arrow) and nsp1a/3-dependent cleavage sites (cyan arrows), with predicted sizes. (**B and D**) Immunoblots of lysates from HEK293T cells transfected with the indicated GFP-nsp1a-V5 expressing plasmids and probed for GFP, V5, and GAPDH. Cyan arrows indicate predicted end-product proteins, and gray arrows indicate predicted size-based intermediates. (**E**) Immunoblot of lysates from HEK293T cells co-transfected with GFP-nsp1a∆655-V5 plasmids (WT and indicated mutants described in the text) or co-transfected with empty plasmid or GFP-nsp1a∆655-HA (last lane) plasmids and probed for V5, HA, and GAPDH. Annotated gray and cyan arrows represent predicted protein products based on molecular weight. * Gray arrows represent potential aggregates or higher-order complexes due to the absence of heat during sample preparation.

Using these expression plasmids, we sought to confirm the previously predicted cleavage sites via site-directed mutagenesis of these residues. First, we introduced a V409A substitution at the predicted nsp1a/2 and nsp1a/3 junction in a GFP-nsp1a∆655-V5 expression plasmid (Fig. 4A). Comparison of lysates from cells expressing WT vs V409A GFP-nsp1a∆655-V5 showed no difference in generation of the ~28 kDa nsp1a/3-dependent cleavage product, suggesting that Val 409 is not involved in cleavage at the junction between nsp1a/2 and nsp1a/3 (Fig. 4B) (21, 25–27).

Additionally, mutation of the other two predicted residues, Gln 567 and Glu 654, at the cleavage site between nsp1a/3 and nsp1a/4 was introduced into a GFP-nsp1a-V5 expression plasmid (Fig. 4C). Based on the crystal structural of nsp1a/3, Gln 567 resides within the substrate-binding site, suggesting it is not likely a site for proteolysis (32). To confirm this, we generated a Q567A substitution construct and observed no difference in nsp1a processing in the production of a ~40 kDa protein band compared to the WT construct (Fig. 4D). Next, we assessed the effect of an E654A substitution in nsp1a processing. We found that this substitution abolished nsp1a processing, similar to the S551A-inactivated protease, suggesting an essential role of this residue for cleavage. To further assess whether Glu 654 is a required residue in a potential cleavage motif or serves an alternate function in protease activity, we co-expressed GFP-nsp1a-E654A-V5 with WT GFP-nsp1a∆655-HA. Co-expression with the WT protease rescued processing of nsp1a-E654A, suggesting that this residue likely is not involved in a potential cleavage motif and may have an alternate role in polyprotein processing or protease activity (Fig. 4E).

### Identification of the nsp1a/2 and nsp1a/3 protease cleavage site

**Fig 5:**
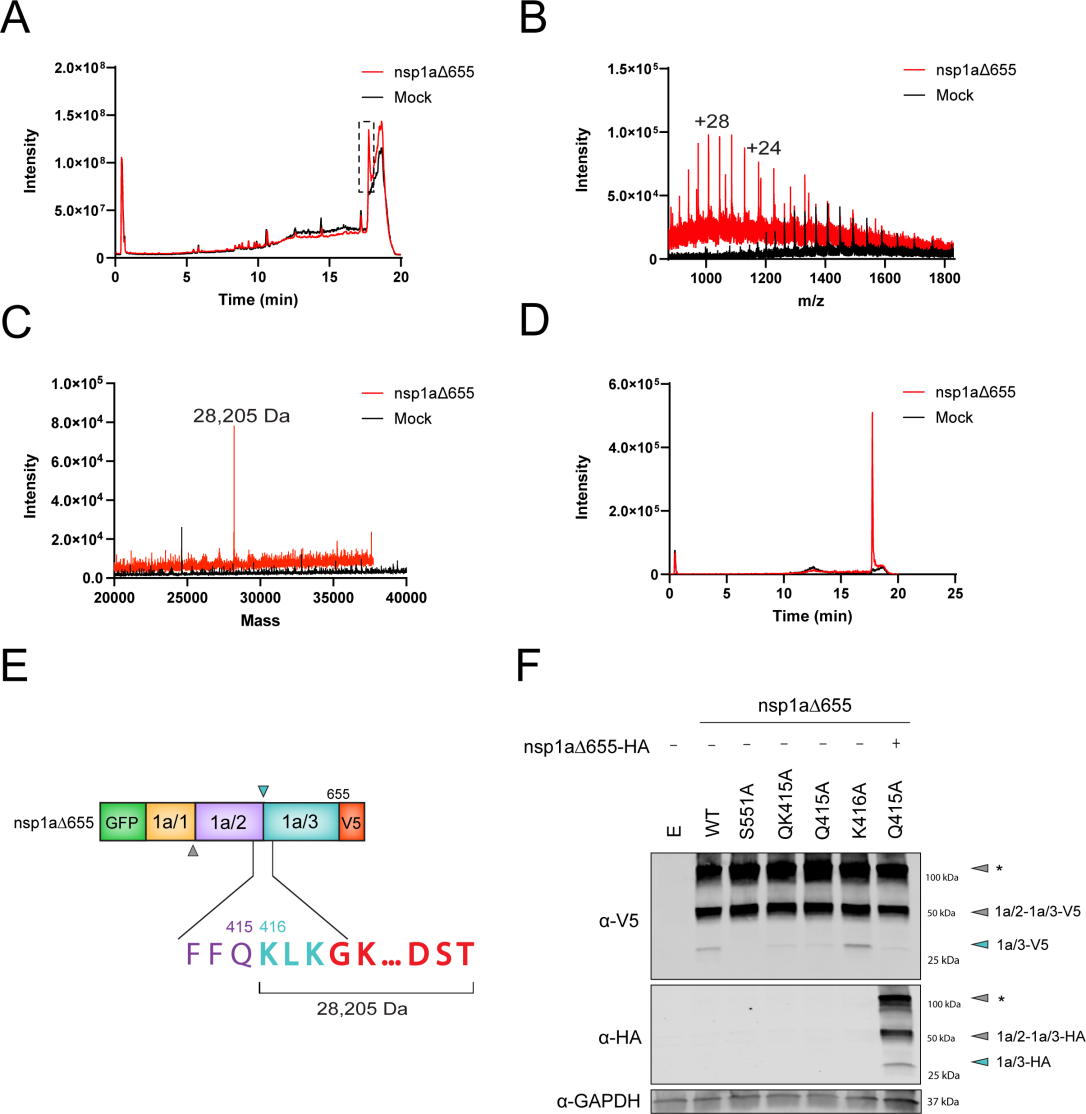
Identification of the nsp1a/2 and nsp1a/3 protease cleavage site using mass spectrometry. (**A**) Total ion current (ToF) data for V5 tag purified samples from non-transfected (mock, black) and GFP-nsp1a∆655-V5-transfected HEK293T cell lysates (red). Dashed black box highlights the elution peak selected for analysis in panel B. (**B**) ESI-ToF spectra of mock and transfected samples during the elution period highlighted in panel A. (**C**) Deconvoluted spectrum of ion series shown in panel B. (**D**) Extracted ion chromatogram for m/z 1008.4 (+28) for transfected (red) and mock (black) samples. (**E**) Linear model showing the protein sequence that would yield a 28,205 Da protein. (**F**) Immunoblot of lysates from HEK29T cells co-transfected with the indicated GFP-nsp1a∆655-V5 constructs co-transfected with GFP-nsp1a∆655-HA and probed for V5, HA, and GAPDH antibodies. Cyan arrows indicate predicted end-product proteins, and gray arrows indicate predicted size-based intermediates. * Gray arrows represent potential aggregates or higher-order complexes due to the absence of heat during sample preparation.

To identify the cleavage site between nsp1a/2 and nsp1a/3, HEK293T cells were mock-transfected or transfected with the GFP-nsp1a∆655-V5 construct. The cleaved C-terminus protein was immunoprecipitated using V5-trap beads and subjected to reverse-phase LC-ESI-ToF mass spectrometry. A strong elution peak was observed at 17.7 minutes that was not present in the mock-transfected group (Fig. 5A). Examination of the spectra composing this peak revealed a charge state series indicative of a protein (Fig. 5B). Maximum entropy analysis of the charge state series yielded a mass of 28,205 Da (Fig. 5C). The extracted ion chromatogram for the +28 ion (m/z = 1008.4) shows this protein to be unique to the transfected cells (Fig. 5D). The experimental mass agrees with the mass of a protein beginning at Lys 416 and ending at the V5-epitope tag, as determined by MS-nonspecific analysis (Fig. 5E), suggesting the cleavage between nsp1a/2 and nsp1a/3 occurs at the junction of Gln 415 and Lys 416.

To better understand the roles of Gln 415 and Lys 416 in nsp1a/3-dependent cleavage, we performed mutagenesis of these residues in the GFP-nsp1a∆655-V5 expression plasmid. Immunoblots of lysates transfected with WT showed production of the expected ~28 kDa nsp1a/3-V5 product; however, a dual substitution of Gln 415 and Lys 416 (QK415A) demonstrated robust restriction of nsp1a/3-V5 production similar to that of an S551A catalytically inactivated protease (Fig. 5F). To further evaluate this junction, we mutated Q415A and K416A individually and observed that Q415 is critical for protease cleavage, as mutagenesis blocks nsp1a/3-V5 production, whereas K416 is dispensable. Furthermore, the production of nsp1a/3-V5 could not be rescued by co-expression of Q415A with WT GFP-nsp1a∆655-HA, suggesting that this residue is involved in cleavage at this junction (Fig. 5F). Together, these data demonstrate that Q415 represents the P1 position of the cleavage site between nsp1a/2 and nsp1a/3.

### Identification of a consensus cleavage motif within the HAstV polyprotein

**Fig 6:**
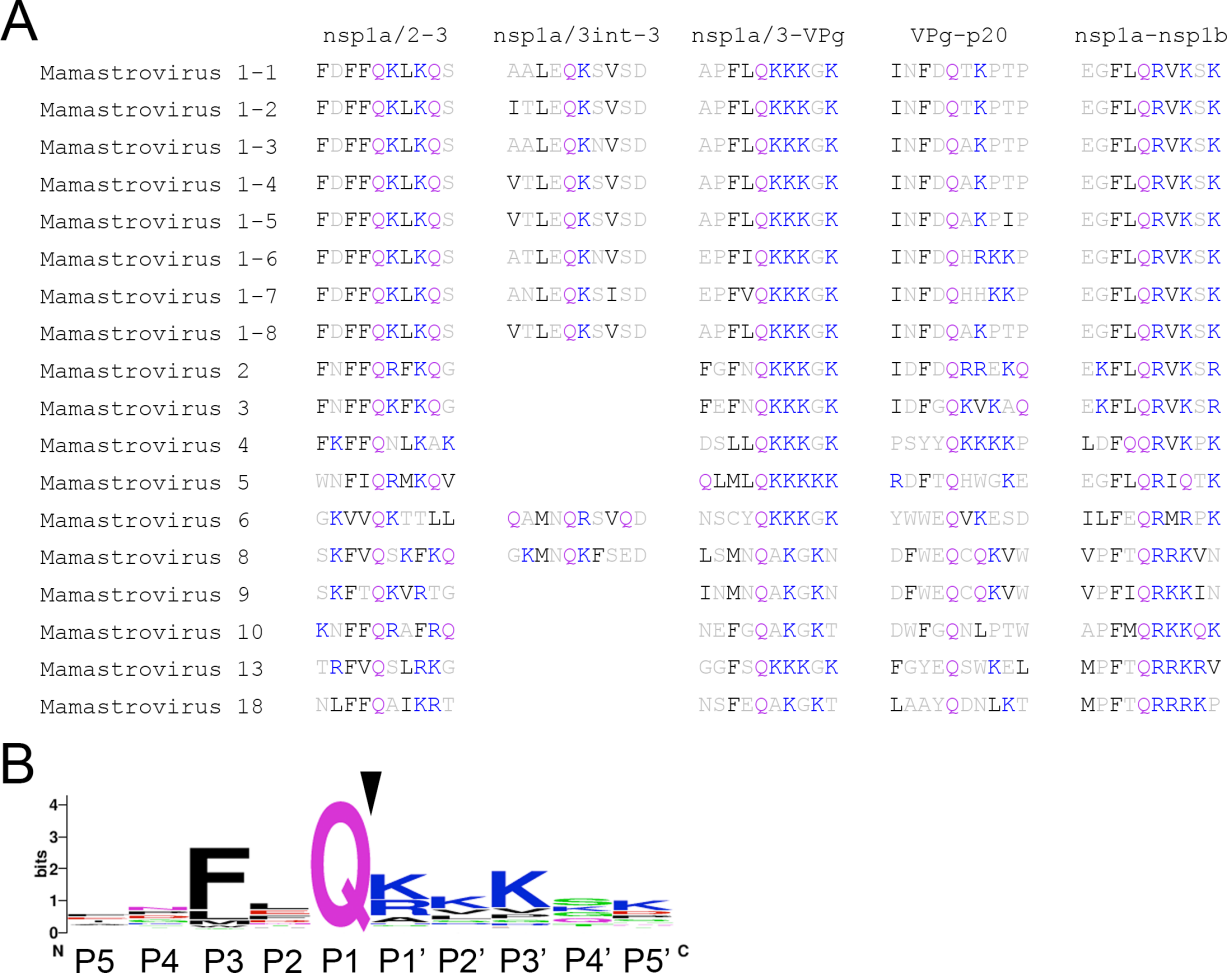
Identification of a consensus cleavage motif. (**A**) Sequence alignment of P5 to P5′ sequences from the indicated 18 MAstV sequences at validated HAstV1 (MAstV1-1, NC_001943.1) cleavage sites. Gln, purple (P1 position); hydrophobic residues, black; basic residues, blue. The absence of sequence indicates no conserved sequence identified at that site. (**B**) Consensus sequence of P5 to P5′ within MAstVs showing a ϕ – X – Q ↓ motif, followed by at least one basic residue in the P1′–P3′ sites.

To determine if the polyproteins of multiple HAstV species have a conserved cleavage motif, we evaluated the sequences of 18 MAstV isolates surrounding the Q415/K416 region. This led to the identification of a conserved di-hydrophobic residue (ϕ) – Q – K/R motif near predicted junctions of nsp1a/2 and nsp1a/3 (Fig. 6A). Using this sequence, we scanned the HAstV1 polyprotein for this motif at regions that would result in the generation of products corresponding to the observed V5-tagged products of ~40 kDa (i), ~37 kDa (ii), and ~25 kDa (iii) protein sizes (Fig. 3B). With this approach, we identified a ϕ – X – Q motif followed by at least one basic residue within the P1′–P3′ sites at potential cleavage junctions of the viral polyprotein (Fig. 6B).

**Fig 7:**
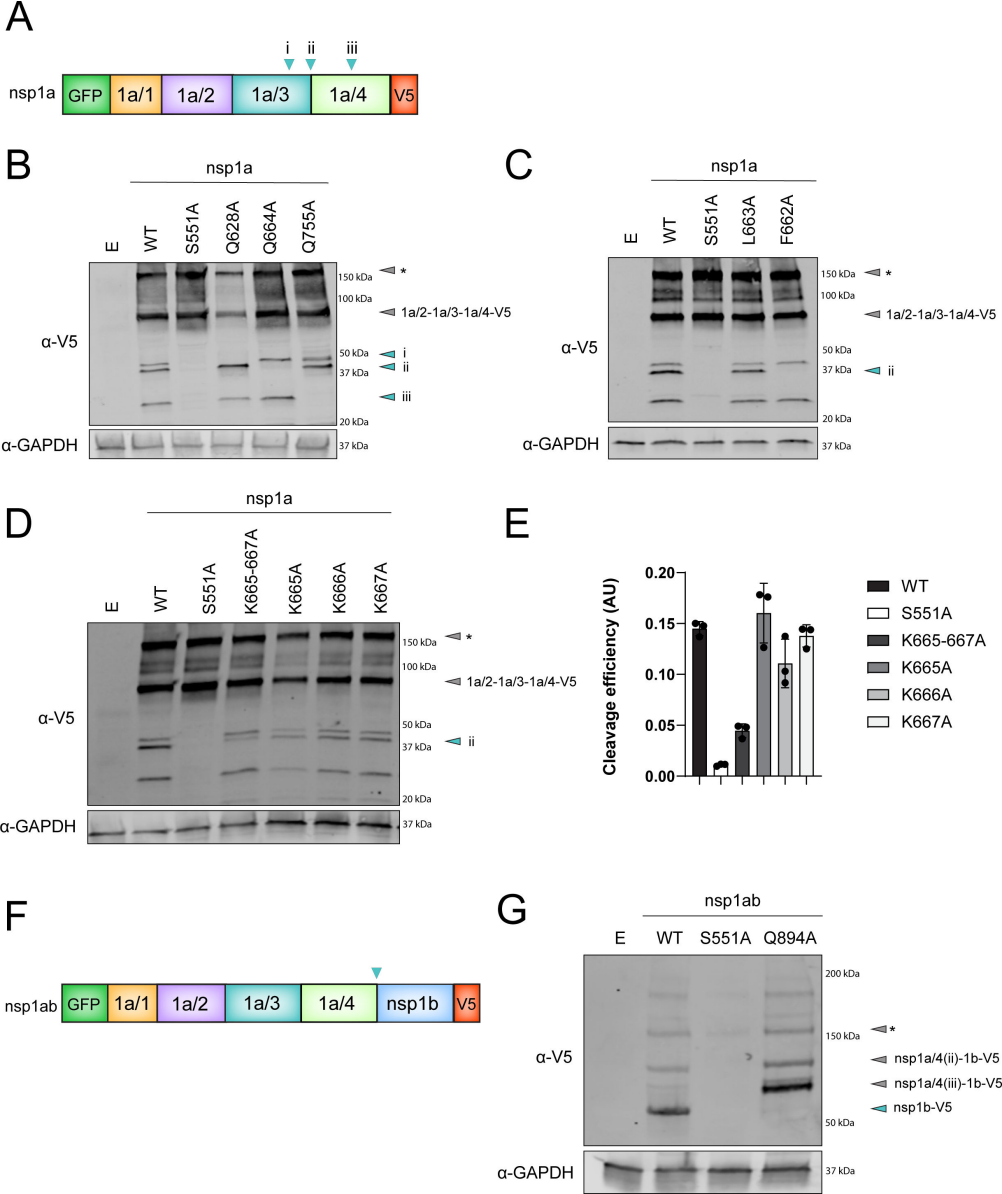
Mutational analysis of the consensus motif. (**A and F**) Linear models of dual-tagged nsp1a (**A**) and nsp1ab (**F**). (**B, C, D and G**) Immunoblots of lysates from HEK293T cells transfected with the indicated WT and indicated mutant plasmids. Immunoblots were probed for V5 and GAPDH. Annotated cyan arrows represent cleavage products indicated in (**A and F**). Annotated gray arrows represent predicted intermediate polyproteins. * Gray arrows represent potential aggregates or higher-order complexes due to the absence of heat during sample preparation. (**B**) Extended immunoblot from Fig. 3B, showing site-specific restriction of nsp1a processing. (**E**) Quantification of immunoblots represented in (**D**). Data represent the average ± SD intensity ratios in arbitrary units (AU) of the final cleavage product of nsp1a/4(ii)-V5 (cyan arrow) signal to full V5-signal within the lane, normalized to GAPDH intensity, and individual data points of three independent experiments are shown in filled circles.

To confirm the presence of a conserved nsp1a/3 cleavage motif within the HAstV polyprotein, we introduced individual mutations within the GFP-nsp1a-V5 construct to the proposed P1 sites at two sites between nsp1a/3 and nsp1a/4, as well as an internal nsp1a/4 site. These proposed P1 sites included Gln 628 (i), Gln 664 (ii), and Gln 755 (iii) (Fig. 7A). We found that alanine substitution of each of these residues abolished the production of a specific nsp1a-V5 product (Fig. 7B), suggesting that the conserved Gln at these junctions serves as the P1 position in the protease cleavage motif. To further characterize the proposed cleavage motif, we mutated the hydrophobic residues at the P2 and P3 position at the nsp1a/4 (ii) site. Alanine substitution of the P2 position (L663A) did not alter production of the ~37 kDa band; however, the substitution of the P3 position (F662A) resulted in loss of this band, suggesting that it is an essential residue for cleavage at this junction (Fig. 7C). Additionally, the substitution of the lysine residues at P1′–P3′ at the nsp1a/4 (ii) site decreased, but did not abolish, the production of the ~37 kDa product. This suggests that these residues are not essential for cleavage but may facilitate interactions with the substrate-binding pocket of nsp1a/3 (Fig. 7D and E).

Our data demonstrate that cleavage between nsp1a and nsp1ab is mediated by nsp1a/3 (Fig. 3B and D). Analysis of the sequence between nsp1a and nsp1b revealed the presence of the identified cleavage motif, which is highly conserved among MAstV sequences (Fig. 6A and B). Alanine substitution at the conserved P1 Gln at this junction (Q894A) in the GFP-nsp1ab-V5 construct blocked production of the ~63 kDa nsp1b-V5-specific cleavage product and resulted in accumulation of a ~79 kDa product that likely corresponds to nsp1a/4(iii)−1b-V5 (Fig. 7G). Together, these experiments have identified a consensus cleavage sequence for nsp1a/3-mediated polyprotein processing in HAstV1.

### Cleavage at conserved motifs is essential for infection

**Fig 8:**
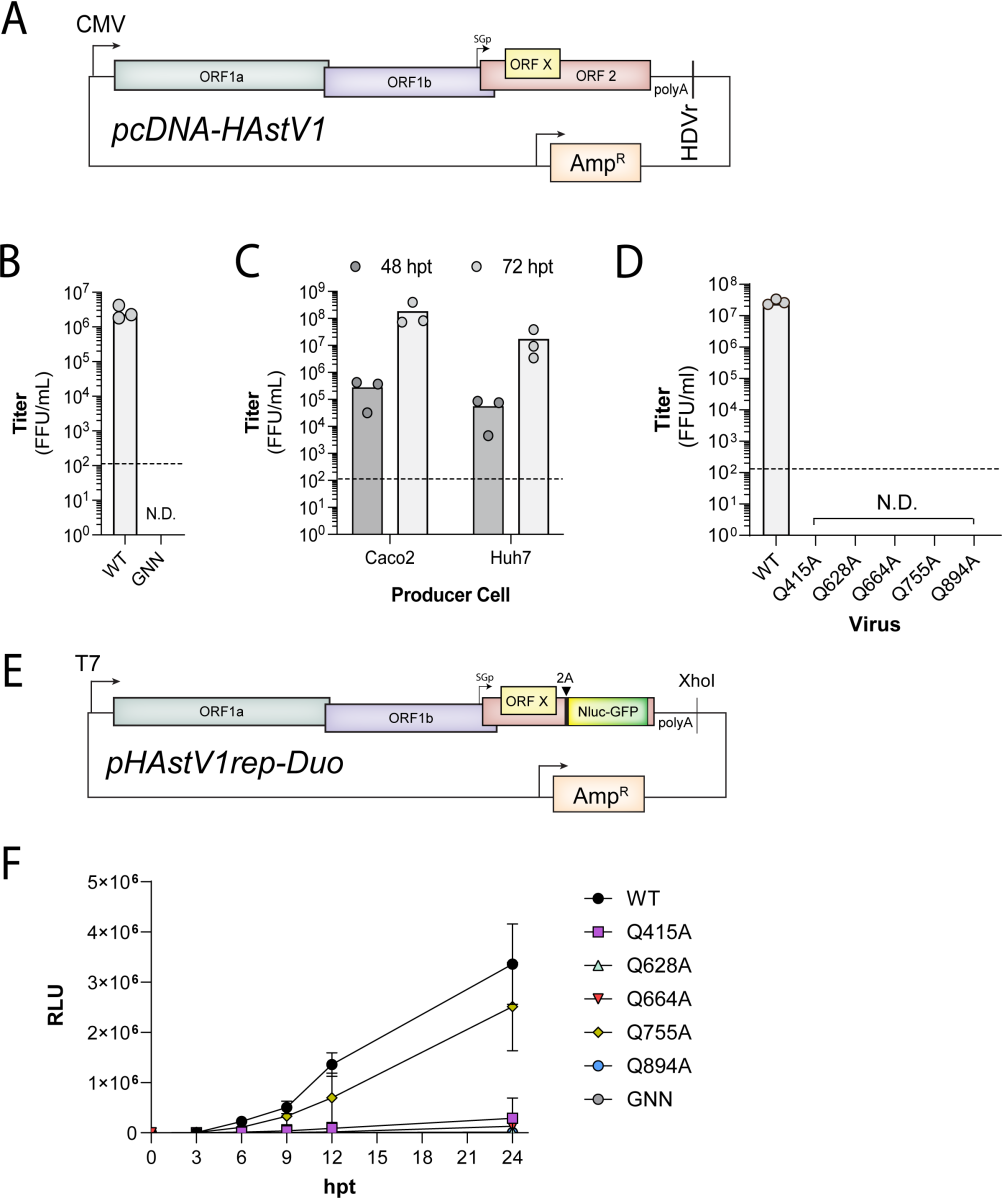
Impact of conserved Glutamine substitution on infection and replication. (**A**) Schematic of the pcDNA-HAstV1 infectious clone. The viral genome with a polyA_16_ is expressed from a CMV promoter (CMV), and the 3′-end followed by a hepatitis delta virus ribozyme (HDVr) sequence. (**B**) Titer of WT HAstV1 and polymerase dead (GNN) HAstV1 virus recovered from pcDNA-HAstV1 transfected cells. Data are shown as the average ± SD focus-forming units per milliliter (FFU/mL) representative of three independent experiments. (**C**) Titer of WT HAstV1 virus recovered from pcDNA-HAstV1 transfected Huh7 or Caco2 at the indicated time points. Data are shown as FFU/mL of three independent experiments. (**D**) Titers of WT and indicated mutants. (**B–D**) Dashed line indicates the limit of detection for the assay. N.D., not detected. (**E**) Schematic of T7-driven pHAstV1rep-Duo replicon. 2A, porcine teschovirus 2A skipping peptide. (**F**) Luciferase activity in lysates from BHK-21 cells transfected with the indicated pHAstV1rep-Duo WT and mutant replicon plasmid-derived RNAs at indicated hpt. Data are shown as the relative light units (RLUs) as the average ± SD, representative of three independent experiments.

To further determine the impact of Gln at the P1 position throughout the polyprotein of HAstV1 in regulation of virus infection, we generated a tractable DNA-launched infectious clone (pcDNA-HAstV1). This plasmid consists of a CMV promoter-driven HAstV1 genome followed by polyA_16_ and HDVr sequence to maintain the integrity of the 3′ end (Fig. 8A). Titration of virus present in the supernatants of transfected Huh7 and Caco2 cells demonstrated robust recovery of infectious HAstV1 in both cell types at 72 hpt with no virus detected in cleavage site mutants and the polymerase dead mutant (GDD/GNN mutations in RdRp; Fig. 8B and C). Using this pcDNA-HAstV1 infectious clone, we introduced individual mutations to the P1 sites, Gln 415, Gln 628 (i), Gln 664 (ii), Gln 755 (iii), and Gln 894, within the HAstV1 polyprotein cleavage junctions. Our results show that viruses containing Gln to Ala substitutions were unable to be rescued (Fig. 8D), further demonstrating the importance of these residues for polyprotein processing during infection.

Next, we sought to determine the effect of Gln to Ala substitutions at the polyprotein cleavage junctions on viral replication. To do this, we constructed an HAstV1 replicon system (pHAstV1rep-Duo) where we replaced a portion of ORF2 with a 2A-NanoLuciferase (NanoLuc)-GFPzeo fusion protein sequence (Fig. 8E). We then introduced Ala substitutions to Gln 415, Gln 628 (i), Gln 664 (ii), Gln 755 (iii), and Gln 894. Transfection of *in vitro* transcribed RNA resulted in robust NanoLuc activity from both WT and Q755A; however, the remaining mutated constructs failed to yield high levels of NanoLuc activity, similar to the polymerase dead (GNN) mutant (Fig. 8F). These results imply that the production of all individual viral proteins is essential for infection, but cleavage at Q755 is dispensable for replication.

## DISCUSSION

**Fig 9:**
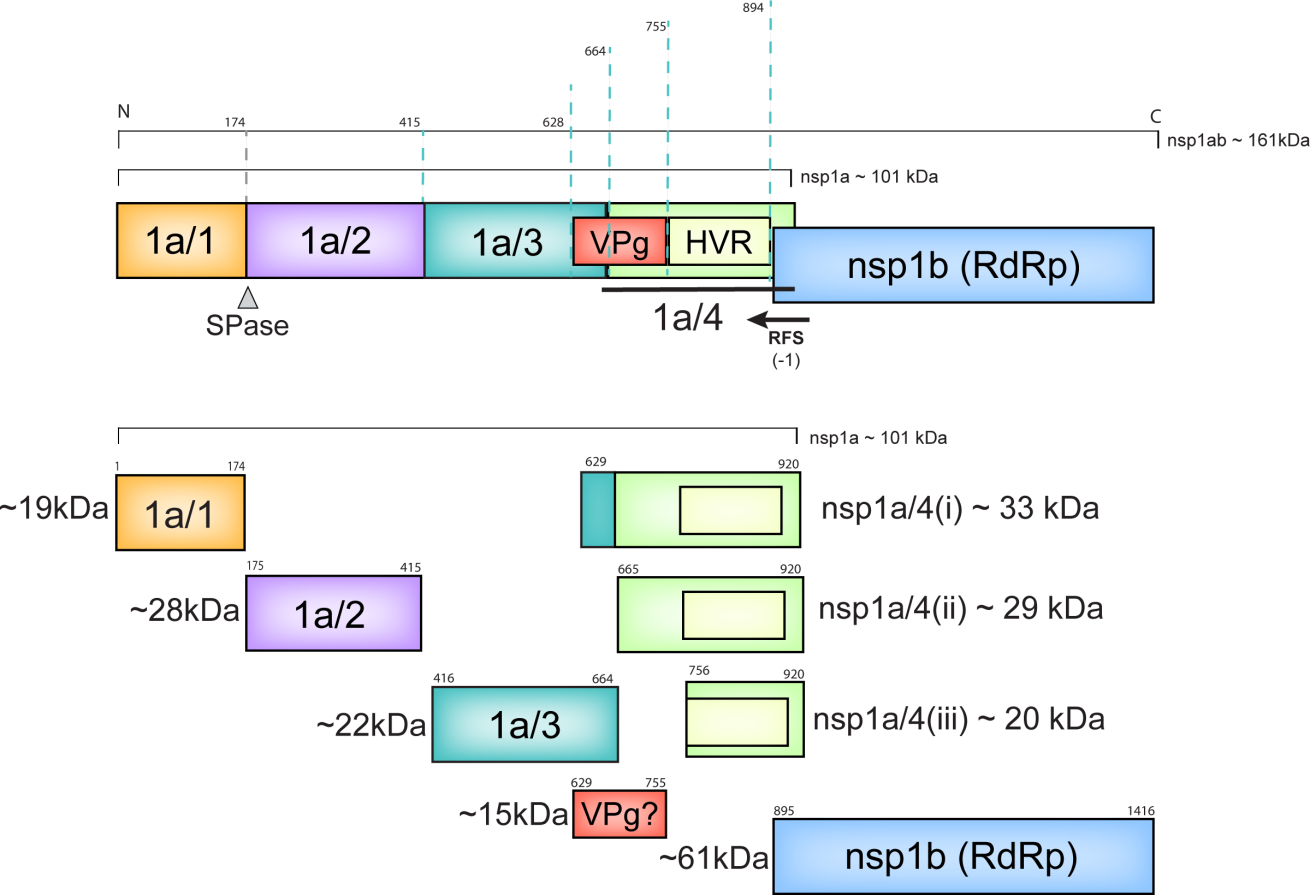
HAstV1 nsp1a/3 targets a consensus motif to mediate cleavage of the viral nonstructural polyproteins. Model of HAstV polyprotein processing, as determined by site-directed mutagenesis. Dashed cyan lines represent experimentally defined cleavage sites. Individual protein products are indicated with the calculated molecular weight from the protein sequence.

Viral polyprotein precursors are processed through cleavage events mediated by host and viral proteases to produce the functional subunits required for infection (41–45). In the case of astroviruses, which encode two nonstructural polyproteins, previous reports have indicated the viral protease, nsp1a/3, cleaves all cytoplasmic junctions at undefined sites (25, 27, 46). Here, we show that HAstV1 nsp1a/3 targets a conserved motif to mediate cleavage of viral nonstructural polyproteins, which includes the production of stable intermediates (Fig. 6 and 9). Furthermore, we show that efficient polyprotein processing is essential for viral replication and production of viral progeny. However, processing of nsp1a/4-VPg is dispensable for viral replication, suggesting that cleavage of this functional intermediate is required for events downstream of viral replication (Fig. 9). Collectively, our results provide essential information regarding the precise HAstV polyprotein processing that will allow for future studies to investigate viral protease activity and define the roles of other individual proteins or functional intermediates during infection.

Previous studies hypothesized that nsp1a/3-mediated cleavages occur at Val 409/Ala 410, Gln 567/Thr 568, and Glu 654/Ile 655 (25, 27). However, these sites were predicted based on the size of products observed on immunoblots or using autoradiography. Furthermore, some of these studies were performed with T7-promoter-driven viral ORFs in cells infected with recombinant vaccinia virus expressing the T7 DNA-dependent RNA polymerase. Therefore, interpretation of these results may be complicated due to vaccinia virus manipulation of the host cell and production of the I7L protease, which may result in aberrant HAstV1 polyprotein cleavage products. Thus, we generated a CMV-promoter-driven system using dual-tagged HAstV1 nonstructural proteins to investigate nsp1a/3-dependent cleavage of the polyprotein. Using these molecular tools, we were able to identify nsp1a/3-dependent cleavage products. Despite the accumulation of precursor proteins with these tools, we were able to consistently reproduce cleavage products that could be abolished through mutagenesis. It is possible that low cleavage efficiency observed for some products is a mechanism required for the production of functional protein intermediates; however, low cleavage efficiency observed during overexpression may not be representative of polyprotein processing in the context of virus-induced remodeled membranous structures. The proteins involved in host membrane remodeling have yet to be defined, partially due to the lack of information regarding polyprotein cleavage sites. Importantly, this issue has been resolved by identification of a conserved nsp1a/3 cleavage sequence (ϕ – X – Q↓) through mass spectrometry and subsequent validation via site-directed mutagenesis analysis of polyprotein expression plasmids.

Despite the conservation of at least one basic amino acid in the P1′–P3′ region, our data suggest that the presence of basic residues downstream of the cleavage site is not required for proteolysis but promotes cleavage efficiency. Analysis of the previously published structure of the HAstV1 protease domain shows a negative polarity patch of residues adjacent to the catalytic site (32). Thus, it is possible that a net positive charge downstream of the P1 Gln in the substrate may facilitate viral cleavage efficiency during infection but is not absolutely required for cleavage activity. Despite the variability downstream of the Gln at the P1 site, it is evident that the presence of a conserved hydrophobic residue at the P3 site is essential for proteolysis by nsp1a/3 (Fig. 7).

Interestingly, the truncated protease domain (aa432-587 and aa432-655) previously utilized for protein X-ray crystallography and biochemical studies, which lacked the residues 415–432 and a region of a predicted coiled-coiled domain at the C terminus of nsp1a/3, was unable to cleave peptides containing the consensus motif defined above (32). This discrepancy suggests that there are proteolytic determinants outside of the substrate consensus motif and the catalytic domain of the viral protease that are necessary for cleavage activity. However, it is unknown if these additional domains are present within the regions of nsp1a/3 that were truncated to promote protein stability or if they are within another domain of the polyprotein. A similar mechanism has been seen in other +ssRNA viruses, such as flaviviruses and hepatitis C virus (47, 48). These viruses require additional nonstructural protein domains outside of the viral protease to facilitate cleavage activity (48, 49). This highlights the importance of identifying additional proteolytic determinants required for catalytic activity to further understand the mechanisms of substrate cleavage by the viral protease, which are critical for the development of targeted antivirals.

A previous study identified the HAstV8 VPg protein to be ~13–15 kDa, which is larger than those of the *Picornaviridae* but consistent with those of the *Caliciviridae* (50). In this study, the sequence of VPg was predicted to be amino acids 665–755, which would yield a ~10.8 kDa protein that is smaller than the experimentally observed VPg protein. The discrepancy in size led us to identify the internal nsp1a/3 cleavage site at Q628. Thus, our data suggest that the HAstV1 VPg protein is produced from cleavage at an internal site within the C-terminus of nsp1a/3 and an internal site of nsp1a/4 (Q755) that yields a protein predicted to be ~15 kDa (Fig. 9), which is consistent with what has been previously described (34, 35). Sequence analysis of non-classical human strains of MAstVs indicates variability within this internal nsp1a/3 site (Fig. 6A). However, in the absence of internal nsp1a/3 cleavage, the predicted size of VPg among non-classical MAstVs remains ~10–15 kDa, but the size of these proteins has yet to be shown experimentally.

Previous studies have shown that the nsp1a/4-derived p20 product is a phosphoprotein that localizes to the ER and colocalizes with viral RNA; however, the specific role of this protein during infection has yet to be investigated (33, 51, 52). Additionally, there appear to be several stable intermediates of nsp1a/4, which may perform specific functions during infection. Proteolytic processing of some RNA viruses, such as enteroviruses and flaviviruses, leads to the production of functional intermediates with diverse functions from fully processed proteins (53, 54). Thus, it will be important to utilize the polyprotein processing data presented here to facilitate future studies investigating the roles of specific polyprotein cleavage products during infection.

Using reverse genetics, we have shown that blocking polyprotein cleavage abolishes recovery of progeny virus. However, the restriction of VPg production via alanine substitution of Q755 allowed for substantial replication. This finding is consistent with previous enterovirus reports that indicate VPg-containing polyprotein intermediates are capable of mediating replication (55). Thus, the efficiency and temporal processing of the HAstV polyprotein warrants further investigation to understand the importance of individual and intermediate products during infection.

Overall, this study uncovered the cleavage recognition motif targeted by the HAstV1 serine protease, which advances understanding of the complexity of viral polyprotein processing. Though functions for nsp1a/3 (serine protease), VPg, and nsp1b (RdRp) have been characterized, the function(s) of the remaining nonstructural proteins within the viral polyprotein are still unknown, largely due to a poor understanding of their specific sequences. However, the results of this study have allowed us to determine the N- and C-termini of each viral nonstructural protein and their cleavage intermediates, which will be essential for future studies focused on identifying the role(s) of each functional viral protein. Furthermore, these results can help facilitate the development of biochemical assays to look at the cleavage activity of nsp1a/3, which is critical for the development of targeted antiviral interventions.

## Data Availability

Data will be made available upon reasonable request to the corresponding author.
